# Videoconference-Delivered Acceptance and Commitment Therapy for Family Caregivers of People With Dementia: Pilot Randomized Controlled Trial

**DOI:** 10.2196/67545

**Published:** 2025-03-31

**Authors:** Areum Han, Robert Oster, Hon Yuen, Jeremy Jenkins, Jessica Hawkins, Lauren Edwards

**Affiliations:** 1 Department of Occupational Therapy University of Alabama at Birmingham Birmingham, AL United States; 2 Division of Preventive Medicine University of Alabama at Birmingham Birmingham, AL United States; 3 Telehealth Private Practice Billings, MT United States; 4 Arts in Medicine University of Alabama at Birmingham Birmingham, AL United States

**Keywords:** acceptance and commitment therapy, Alzheimer disease, caregivers, dementia, depression, web-based intervention, quality of life, randomized controlled trial, stress, videoconferencing

## Abstract

**Background:**

Family caregivers of individuals with dementia face significant mental health challenges. Acceptance and commitment therapy (ACT) has emerged as a promising intervention for improving these caregivers’ mental health. While various delivery modes of ACT have been explored, there is a need for evidence on the efficacy of videoconference-delivered ACT programs for this population.

**Objective:**

This pilot randomized controlled trial, conducted in the United States, aims to assess the effects of a videoconference-delivered, therapist-guided ACT program on reducing depressive symptoms and improving other mental health outcomes among family caregivers with depression who give care to individuals with dementia, compared to a control group that received psychoeducation materials only.

**Methods:**

This 2-arm, parallel-group pilot randomized controlled trial randomly assigned 33 family caregivers to either a 10-week videoconference-delivered ACT program (n=16, 48%) or a control group that received psychoeducation materials alone (n=17, 52%). Depressive symptoms (primary outcome) were measured using the Patient Health Questionnaire-9. Secondary outcomes included anxiety, stress, psychological quality of life (QoL), caregiver burden, predeath grief, guilt, and ACT process measures. Outcomes were assessed in the pretest, posttest (10-12 weeks after pretest), and a 3-month follow-up (3 months after posttest, approximately 5-6 months after pretest). An intent-to-treat approach was used for all outcome analyses. Linear mixed-effects models for repeated measures were used to analyze outcomes.

**Results:**

The ACT group reported significantly greater improvements in stress (*P*=.043) and psychological QoL (*P*=.014) in the posttest compared to the control group. Within the ACT group, participants experienced a significant decrease in depressive symptoms, with a mean (SE) change of –6.09 (1.16) points (95% CI –8.42 to –3.76; *P*<.001) in the posttest and –6.71 (1.45) points (95% CI –9.63 to –3.81; *P*<.001) in the 3-month follow-up. These changes exceed the estimated minimal clinically important difference on the Patient Health Questionnaire-9. In addition, the ACT group reported significant improvements in anxiety, stress, psychological QoL, caregiver burden, predeath grief, guilt, values-driven action, and experiential avoidance at both posttest and 3-month follow-up. A sensitivity analysis, excluding 1 participant with near-outlier data, revealed statistically significant between-group differences in depressive symptoms at posttest (*P*=.037); stress at posttest (*P*<.001) and in 3-month follow-up (*P*=.001); psychological QoL at posttest (*P*<.001); caregiver burden at posttest (*P*=.003) and in 3-month follow-up (*P*=.003); predeath grief in 3-month follow-up (*P*=.031); and values-driven action at posttest (*P*=.032).

**Conclusions:**

The videoconference-delivered ACT program showed promise in improving mental health outcomes and ACT processes among family caregivers with depression who give care to individuals with dementia. Future studies should aim to replicate these findings with larger, more diverse caregiver populations and explore the long-term efficacy of videoconference-delivered ACT programs.

**Trial Registration:**

ClinicalTrials.gov NCT05043441; https://clinicaltrials.gov/study/NCT05043441

## Introduction

In the United States, around 11 million individuals offer unpaid care to individuals with Alzheimer disease and related dementias (ADRD), with approximately two-thirds identifying as women [1]. Most of these caregivers are adult children caring for their parents, with spouses making up the next largest group [1]. These family caregivers provide critical support for individuals with ADRD, helping their relatives age in place [2,3]. Therefore, maintaining these caregivers’ health is imperative; however, they face significant unmet mental health needs [4]. Recent meta-analyses estimate that approximately 34% of these family caregivers experience depressive symptoms, and 32.1% experience anxiety [5,6]. The demands of caring for individuals with ADRD often prevent caregivers from engaging in self-care and other personally valued activities and can lead to significant caregiver burden, stress, and reduced quality of life (QoL) [5,7-9]. Psychoeducation and skill-building interventions have been the predominant approaches to addressing these caregivers’ needs, but meta-analyses indicate that these interventions alone do not significantly improve caregivers’ mental health, particularly in alleviating depressive symptoms [10]. In contrast, psychoeducational interventions with psychotherapeutic components, as well as psychotherapy and counseling, have shown small effects in reducing depressive symptoms of these caregivers [10].

Acceptance and commitment therapy (ACT) is an evidence-based, transdiagnostic approach that combines acceptance and mindfulness with behavior change processes to support living in alignment with personal values [11,12]. ACT is grounded in the psychological flexibility model, which encompasses six core processes: (1) acceptance of unwanted thoughts and emotions; (2) cognitive defusion, or detachment from unhelpful thoughts and emotions; (3) present-moment awareness; (4) self-as-context, or seeing oneself as the observer of thoughts and feelings; (5) values clarification; and (6) establishing patterns of behavior that align with values [12]. ACT skills training can help family caregivers manage the difficult thoughts and emotions arising from their caregiving roles, such as depression, anxiety, stress, guilt, and grief while managing caregiving responsibilities alongside other important areas of life [13,14].

To our knowledge, there have been 11 intervention studies investigating ACT for family caregivers of individuals with ADRD, most of which were published within the last 4 years [14-24]. These studies were conducted in Spain [14-17], the United States [18-21], the United Kingdom [22], Germany [23], and the Netherlands [24]. They demonstrated the acceptability and benefits of ACT in improving depressive symptoms, anxiety, stress, caregiver burden, guilt, grief, and ACT processes (eg, experiential avoidance and values-driven action) [14-24]. However, most of these studies were preliminary, with half using one-group pretest-posttest designs as feasibility and pilot studies [17,18,20-22,24], and the rest using randomized controlled trials (RCTs) conducted on relatively smaller scales or resulting in relatively smaller sample sizes at follow-ups [14-16,19-23] compared to typical RCTs. For example, an RCT conducted in Spain randomized 135 caregivers into in-person ACT, in-person cognitive behavioral therapy, and a control condition, and 6-month follow-up data were analyzed for 55 participants in total (25 in the ACT group) [15]. Most importantly, delivery modes varied across studies, including in-person programs [14-17], telephone-based programs [21,23], and internet-delivered programs, such as coach-guided videoconferences conducted by our study team [18,19] and self-help web modules [20,22,24]). More research is needed to understand the effects of ACT delivered in various delivery modes and across different countries for family caregivers of individuals with ADRD. Intervention effects may vary due to cultural influences on caregiving experiences, coping styles, acceptability, and the suitability of different delivery modes based on caregivers’ characteristics, including race or ethnicity, preferences, and needs [25].

Web-based delivery of ACT could be an effective alternative to in-person services, enhancing accessibility and scheduling flexibility for caregivers who cannot leave their relatives unattended [26-28]. In particular, meta-analyses of ACT across different populations suggest that therapist-guided, web-based programs have greater effects on depressive symptoms and ACT process measures than unguided programs [26,28]. In addition, the use of a self-help approach alone is limited to individuals with mild depressive symptoms, as it is not recommended for those with more severe symptoms [29]. Individually and remotely delivered ACT programs with therapist guidance can provide higher engagement for these caregivers compared to other internet delivery modes [30]. Therefore, videoconference-delivered, therapist-guided programs may be particularly beneficial for family caregivers who face significant mental health challenges, including depressive symptoms, and require one-on-one real-time interaction with a therapist but struggle to attend in-person therapy sessions [18,19].

This study aimed to assess the effects of a videoconference-delivered, therapist-guided ACT program on mental health outcomes and ACT processes in family caregivers with depression who give care to individuals with ADRD, compared to a control group that received psychoeducation materials alone conducted in the United States. The study builds on insights gained and findings from 2 smaller-scale videoconference-delivered ACT studies conducted by our team [18,19], which informed the decision to focus on caregivers with depressive symptoms and to modify the outcome measures.

## Methods

### Study Design and Overview

This study used a 2-arm, parallel-group RCT design. Family caregivers of individuals with ADRD were randomly assigned in a 1:1 ratio to either the videoconference-delivered ACT group or the control group that received psychoeducation materials alone. The study was conducted from late March 2022 to mid-June 2024, with data collection and entry spanning approximately 2.2 years, and a recruitment period lasting about 1.7 years.

### Ethical Considerations

This study was reviewed and approved by the institutional review board at the University of Alabama, Birmingham (IRB-300008123) and registered at ClinicalTrials.gov (NCT05043441) before participant enrollment. All procedures adhered to ethical guidelines for human participant research. All participants provided informed consent prior to their involvement in the study. To protect participants’ privacy and confidentiality, all study data were deidentified before analysis. Data were securely maintained and accessible only to authorized research personnel. Participants received a reloadable ClinCard as compensation for their time—up to US $150 for the intervention group for completing sessions and questionnaires, and up to US $90 for the control group for completing questionnaires only.

### Eligibility Criteria

Participants were recruited based on the following inclusion criteria: (1) adults (aged 18 years or older) primarily responsible for the care of a relative with ADRD, (2) reporting at least mild depressive symptoms (Patient Health Questionnaire-9 [PHQ-9] scores≥5 [31]), (3) access to a computer or smartphone with internet connectivity, and (4) ability to provide informed consent. Exclusion criteria included cognitive, physical, or sensory deficits; being a non-English speaker; psychiatric hospitalizations or mental illness diagnoses in the past 2 years; use of antipsychotic or anticonvulsant medications; or plans to place the relative with ADRD in a nursing home within 6 months.

### Target Sample Size

The target sample size was adjusted from 64 to 32 due to challenges in recruiting family caregivers with depression within the funding period. The following power calculation was performed to justify the revised sample size; this was done before study completion and before the statistical analyses were performed, and as such, is considered a priori. This calculation represents the possible between-group effect and its corresponding effect size (Cohen *d*). Assuming that we will compare repeated measurements for group means, that there are 2 groups, 3 time points (ie, that there are 3 measurements obtained for each participant), an SD for depressive symptoms (PHQ-9 score of 5.20) [32], a moderate correlation between measurements on the same participant of 0.50, and 13 participants per group (26 for the study), we will have 80% power to detect a statistically significant difference in change scores for PHQ-9 of 4.67 and greater between the 2 groups at a 0.05 significance level (2-sided). This is equivalent to being able to detect a statistically significant effect size of 0.90 and greater. This result compares favorably with a previous ACT intervention study for family caregivers with depression who give care to individuals with ADRD in Spain, which reported an effect size of 1.17 for reducing depressive symptoms in the in-person ACT group compared to a control group at posttest and included participants with posttest data in the analyses [15]. Our power calculation was performed using nQuery (version 9.4; Statistical Solutions, Ltd). Considering an estimated 16% dropout rate based on a meta-analysis of dropout rates in ACT RCTs [33], the study aimed to enroll at least 32 participants in total (16 per group). This sample size is also consistent with the recommendation of a minimum of 10 participants per group for pilot RCTs [34].

### Procedures

A study flyer containing the project coordinator’s contact information and a link to the eligibility screening survey created in Qualtrics was distributed to directors of Area Agencies on Aging and centers or groups supporting individuals with ADRD and their caregivers (eg, adult day care centers and support groups) across various geographic locations within the United States, including the south (eg, Alabama, Tennessee, Texas), the northeast (eg, New York, New Jersey, Massachusetts), the Midwest (eg, Michigan, Wisconsin, Indiana), and the west (eg, California, Arizona). Potential participants contacted the project coordinator for more information and completed the eligibility screening survey. Printed consent forms and pretest questionnaires were sent by mail to eligible participants who expressed interest in taking part, along with a preaddressed, prestamped envelope for their return.

A co-investigator, who had no direct contact with participants, used a computer-generated randomization sequence to create a list, which was provided to the project coordinator in sequentially numbered, opaque sealed envelopes. After the project coordinator received the signed consent forms and completed the pretest questionnaires, the envelope associated with the participant’s ID number was opened and informed them of their group assignment. Participants were asked to complete follow-up questionnaires roughly 3 and 6 months after completing their pretest questionnaires.

### The ACT and Control Groups

Participants assigned to the ACT group received individual ACT sessions, led by a trained ACT coach (a licensed professional counselor), via Zoom (Zoom Video Communications) videoconferencing for 1 hour per week over 10 weeks. Additionally, a 1-time 1-hour booster session was provided 1.5 months after the completion of the 10th weekly session to facilitate the maintenance of treatment effects [16]. Participants were provided with additional worksheets and web-based ACT resources for continued use until the booster session. The overview of the 10 weekly ACT sessions is presented in Multimedia Appendix 1. The ACT protocol was developed specifically for family caregivers of individuals with ADRD [18,19].

Printed copies of the ACT session worksheets were sent by mail to participants in the ACT group prior to the first session. The program aimed to actively involve participants through in-session learning activities such as worksheets, metaphors, and case scenarios, along with homework assignments. After each session, the coach shared additional web-based ACT resources, like guided video exercises, and provided a summary of the strategies discussed to support practice between sessions.

Regardless of group assignment, supplemental psychoeducation materials were sent to all participants, including video links and hard copies of resources to better meet these caregivers’ needs. These materials aimed to enhance their understanding of dementia (eg, types, stages, and symptoms), care strategies for different stages of dementia, strategies for managing behavioral and psychological symptoms of dementia, and communication techniques. Participants in the control group continued with care as usual and received the same psychoeducation materials provided by the study team. After their participation in the study ended, the control group was given access to web-based ACT resources and offered a free optional 1-hour session with the ACT coach.

### Feasibility Outcomes

The feasibility of recruitment, eligibility, and retention was assessed by tracking the number of potential participants who completed the eligibility screening survey, those who were excluded, those who consented, the number of dropouts, and the time required to recruit the target sample size. Participant adherence to the program was evaluated through session attendance in the ACT group. The ACT coach maintained session logs, which were reviewed by the principal investigator (the first author) to ensure the coach followed the protocol consistently across participants.

### Data Collection and Entry

#### Overview

Data were collected using self-reported questionnaires in the pretest (baseline), posttest (approximately 10-12 weeks after the pretest), and a 3-month follow-up (approximately 3 months after the posttest, or 5-6 months after the pretest) for both groups. The pretest questionnaire included demographic and caregiving-related questions, as presented in Multimedia Appendix 2. The project coordinator periodically contacted participants regarding the completion and mailing of their questionnaires. When the project coordinator received the completed paper questionnaire, she checked for any missing responses. If any were found, the project coordinator called the participant to obtain the missing information. Two undergraduate research assistants, who were unaware of group assignments, handled data entry using Qualtrics. Data entered by each assistant was compared by a co-investigator who was not involved in data analysis to identify and resolve any potential data entry errors and to finalize the dataset before analysis.

#### Primary Outcome Measure

The primary outcome of this study was the PHQ-9, a 9-item self-report tool that assesses the severity of depressive symptoms over the past 2 weeks, rated on a scale from 0 to 3 [31]. Scores range from 0 to 27, with higher scores indicating more severe depressive symptoms. The PHQ-9 categorizes depressive symptom severity as follows: 0-4=no depressive symptoms, 5-9=mild, 10-14=moderate, 15-19=moderately severe, and 20-27=severe [31]. It has demonstrated good internal consistency and test-retest reliability, as well as criterion validity, construct validity, diagnostic validity for major depressive disorder, and sensitivity to change [31]. The minimal clinically important difference (MCID) is estimated to be 5 points on the PHQ-9 scale [35].

#### Secondary Outcome Measures

The Generalized Anxiety Disorder-7 Scale (GAD-7) is a 7-item self-report tool that assesses the severity of anxiety symptoms over the past 2 weeks, rated on a scale from 0 to 3 [36]. Scores range from 0 to 21, with higher scores indicating more severe anxiety symptoms. Cutoff points of 5, 10, and 15 denote mild, moderate, and severe anxieties, respectively [36]. The GAD-7 has shown good internal consistency and test-retest reliability, as well as criterion validity, convergent validity, construct validity, and sensitivity to change [36-39]. The MCID for the GAD-7 is estimated to be 3 or 4 points [39,40].

The Perceived Stress Scale-10 (PSS-10) is a 10-item self-report questionnaire that assesses how unpredictable, uncontrollable, and overloaded individuals perceived their lives to be over the past month, using a scale of 0 to 4 [41,42]. Scores range from 0 to 40, with higher scores indicating greater stress [42]. The scale has shown good internal consistency and test-retest reliability, as well as convergent validity, construct validity, and concurrent validity [43].

The World Health Organization Quality of Life Instrument–Psychological Health component (WHOQOL-BREF–Psych) has 6 items that assess psychological QoL over the past 2 weeks, rated on a scale of 1 to 5 [44]. Scores range from 6 to 30, with higher scores denoting better psychological QoL. The WHOQOL-BREF–Psych component has demonstrated excellent intrarater reliability, interrater reliability, internal consistency, and adequate convergent validity [44].

The Short Version of the Zarit Burden Interview (ZBI) is a 12-item self-report tool that assesses caregivers’ perceived burden, rated on a scale from 0 to 4 [45]. Scores range from 0 to 48, with higher scores indicating higher levels of caregiver burden. Suggested cutoff points are as follows: 0-9=no or little burden, 10-19=mild to moderate burden, and 20-48=high burden [45]. The ZBI has good internal consistency as well as convergent and discriminant validity in caregivers of individuals with ADRD [45,46].

The Marwit-Meuser Caregiver Grief Inventory-Brief Form (MM-CGI-BF) is a 6-item self-report questionnaire that assesses predeath grief in caregivers of individuals with ADRD (ie, losses, including anticipation of future losses), rated on a scale from 1 to 5 [47]. Scores range from 6 to 30, with higher scores indicating greater grief. The MM-CGI-BF has demonstrated good internal consistency, test-retest reliability, and construct validity in caregivers of individuals with ADRD [47].

The Caregiver Guilt Questionnaire (CGQ) is a 22-item self-report measure that assesses feelings of guilt in caregivers, rated on a scale from 0 to 4 [48]. Scores range from 0 to 88, with higher scores indicating greater levels of guilt. The CGQ has good internal consistency and convergent validity in caregivers of individuals with ADRD [48].

#### Measures Related to ACT Processes

The Acceptance and Action Questionnaire-II is a 7-item self-report measure that assesses experiential avoidance (or efforts to control or suppress unwanted thoughts and emotions, even when doing so is ineffective or has negative consequences) and the broader measure of psychological inflexibility, rated on a scale from 1 to 7 [49]. Scores range from 7 to 49, with higher scores indicating greater experiential avoidance. The Acceptance and Action Questionnaire-II has demonstrated good internal consistency and test-retest reliability, as well as concurrent, predictive, discriminant, and incremental validity [49,50].

The Cognitive Fusion Questionnaire-7 is a 7-item self-report tool that assesses cognitive fusion (ie, being so entangled with thoughts that they dictate behavior in inflexible ways), rated on a scale from 1 to 7 [51]. Scores range from 7 to 49, with higher scores indicating greater cognitive fusion. The Cognitive Fusion Questionnaire-7 has shown good internal consistency and test-retest reliability, as well as concurrent and discriminant validity [51,52].

The Engaged Living Scale-9 is a 9-item self-report questionnaire that assesses values-driven action (ie, clarity and engagement with personal values), rated on a scale from 1 to 5 [53]. Scores range from 9 to 45, with higher scores indicating greater values-driven action. The Engaged Living Scale-9 has demonstrated good internal consistency and construct validity [53,54].

The Self-Compassion Scale-Short Form (SCS-SF) is a 12-item self-report questionnaire that assesses self-compassion, rated on a scale from 1 to 5 [55]. Scores range from 12 to 60, with higher scores indicating higher levels of self-compassion. The SCS-SF has shown good internal consistency and test-retest reliability, as well as convergent validity [56].

### Data Analysis

Composite scores, with appropriate reverse coding of the responses for the outcome measures, were computed for analysis. Descriptive statistics were used to summarize participants’ characteristics. Normality checks, including boxplots, stem-and-leaf plots, normal probability plots, and the Kolmogorov-Smirnov test, were performed on continuous variables. All continuous variables were found to be at least approximately normally distributed. All statistical tests were 2-sided, with a significance level of *α*=.05. Statistical analyses were conducted using SAS (version 9.4; SAS Institute, Inc).

To test the equivalence of groups (ACT and control groups) in participants’ demographic and caregiving-related characteristics, as well as outcome measures in the pretest, the chi-square test (or Fisher exact test when the assumptions for the chi-square were not satisfied) was used for categorical variables, and the 2-sample *t* test was used for continuous variables. An intent-to-treat approach was used for all outcome analyses (ie, data from all participants were analyzed as randomized). Three participants in the control group and 2 participants in the ACT group who did not complete the posttest and 3-month follow-up evaluations were included in these analyses.

Outcomes were explored using linear mixed-effects models for repeated measures [57-60]. These models included group (1=control group and 2=ACT group), which is treated as a fixed effect, time point (1=pretest, 2=posttest, and 3=3-month follow-up), which is treated as fixed but where the slopes of the time points are treated as random, and the group by time point interaction (including components for the group × posttest interaction and the group by 3-month follow-up interaction), with a random intercept for participants. All 3 time points were included in our linear mixed effects models. These models were estimated using restricted maximum likelihood, and a first-order autoregressive covariance matrix was assumed. All linear mixed effects models were run using PROC MIXED of SAS, invoking the REPEATED statement. This statement allows us to incorporate all aspects of random variation into a covariance structure (for our data, a first-order autoregressive covariance matrix) associated with the individual participants. Overall *P* values and their associated *F* values are first reported for group, time point, and the group×time point interaction. Next, the estimated mean change from pretest to posttest within each group, the absolute difference in mean change between groups at posttest, the estimated mean change from pretest to 3-month follow-up within each group, and the absolute difference in mean change between groups in 3-month follow-up, along with corresponding SEs and 95% CIs, were reported; for a given variable, all of these results were obtained from the same linear mixed effects model.

Linear mixed effects models (including those for repeated measures) are robust against missing data, as they can accommodate unbalanced data patterns [57-60]. Therefore, all available observations were included in the analyses. For each outcome variable, participants with at least 1 nonmissing outcome measure were included in the analysis. A Cohen *d* type effect size [61] was calculated for between-group effects using the absolute difference of the means between the 2 groups divided by the common SD and for within-group effects using the estimated change in the means between the 2 time points divided by the SD of change. Due to the exploratory and preliminary nature of this pilot RCT study, no adjustment of *P* values was conducted for multiple statistical comparisons of the outcome measures [62].

## Results

### Participant Flow and Feasibility Outcomes

**Figure 1:**
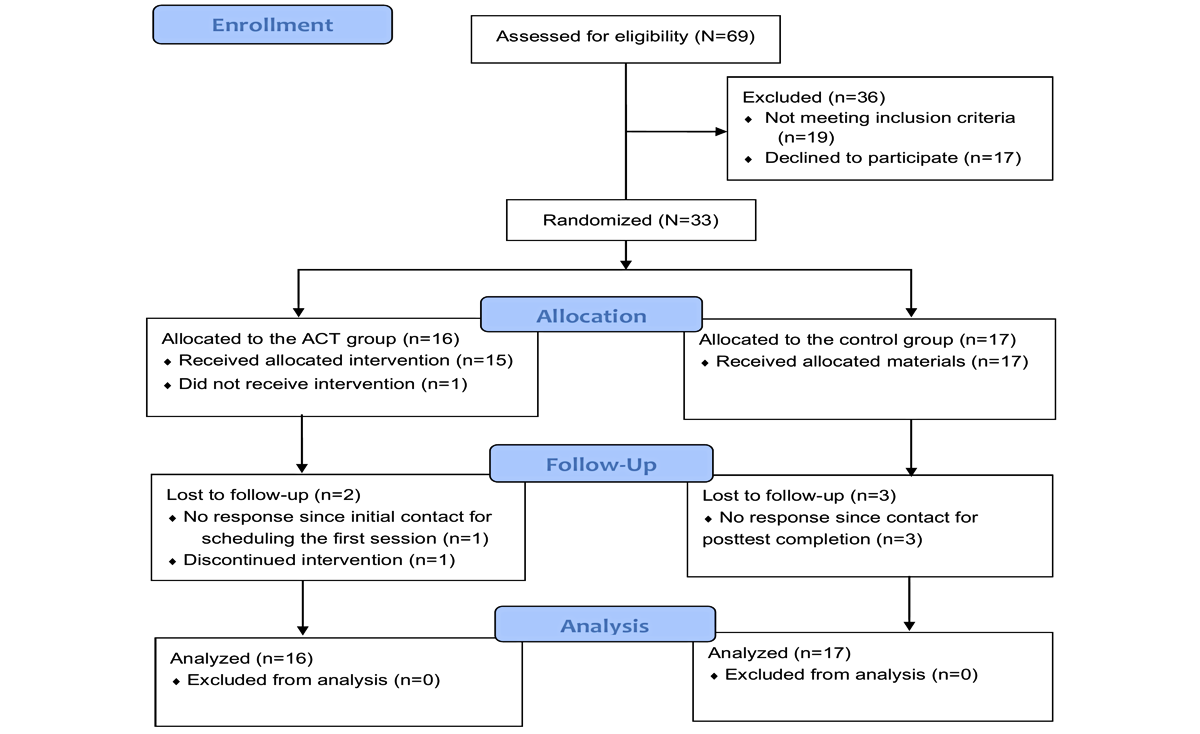
Participant flow diagram of a pilot randomized controlled trial of a videoconference-delivered ACT group and a control group receiving psychoeducation materials, with pretest, posttest, and 3-month follow-up evaluations for depressed family caregivers of individuals with dementia in the United States. ACT: acceptance and commitment therapy.

Figure 1 shows the CONSORT-EHEALTH (Consolidated Standards of Reporting Trials of Electronic and Mobile Health Applications and Online Telehealth) flow diagram for participant recruitment (CONSORT-EHEALTH checklist provided in Multimedia Appendix 3). A total of 69 potential participants completed the study eligibility screening survey over the 1.7-year recruitment period. Of these, 19 did not meet the eligibility criteria, mostly due to not meeting the PHQ-9 screening criteria. Seventeen eligible participants chose not to participate in the study, mostly for unknown reasons, and a few due to concerns about the required language used in the consent form.

The remaining 33 participants gave informed consent, completed pretest questionnaires, and were randomly assigned to either the ACT group or the control group. All participants except 5 (3 in the control group and 2 in the ACT group) completed the posttest and 3-month follow-up questionnaires. Three participants (2 from the control group and 1 from the ACT group) were lost to follow-up after completing the pretest questionnaire and group assignment (no response). One participant in the control group requested withdrawal from the study after the group assignment, and 1 participant in the ACT group requested withdrawal after completing 6 sessions due to a lack of preference for mindfulness practices. All remaining participants in the ACT group completed all 10 weekly ACT sessions and the booster session 1.5 months after the 10th session. The coach adhered to the protocol across participants.

### Baseline Characteristics of Participants

Table 1 presents the characteristics of participants (N=33) and the results of between-group differences in characteristics. Multimedia Appendix 4 presents between-group differences in outcomes at pretest. There were no statistically significant differences between the ACT and control groups in characteristics and outcome measures at pretest. The majority of participants were female (n=29, 88%), non-Hispanic White (n=24, 73%), and daughters of individuals with ADRD (n=21, 64%). Participants’ ages ranged from 23 to 78 (mean 55.2, SD 12.7) years. The time since the relative’s diagnosis of ADRD ranged from 0.4 to 14.2 (mean 4.8, SD 3.8) years.

**Table 1:** Characteristics of participants at pretest in a pilot randomized controlled trial of a videoconference-delivered ACT^a^ group and a control group receiving psychoeducation materials for family caregivers with depression who give care to individuals with dementia in the United States.

		All (N=33)	ACT group (n=16)	Control group (n=17)	Difference between groups, *P* value	
Age (years), mean (SD; range)		55.2 (12.7; 23-78)	54.1 (13.4; 23-78)	56.4 (12.2; 31-78)	.543	
**Sex, n (%)**						≥.99
	Female	29 (88)	14 (88)	15 (88)		
	Male	4 (12)	2 (12)	2 (12)		
**Education, n (%)**						.61
	Postgraduate degree	18 (55)	8 (50)	10 (59)		
	Bachelor’s degree or less	15 (45)	8 (50)	7 (41)		
**Race, n (%)**						≥.99
	Black or Hispanic	9 (27)	4 (25)	5 (29)		
‍	Non-Hispanic White	24 (73)	12 (75)	12 (71)		
**Marital status, n (%)**						.83
	Married or living with a partner	20 (61)	10 (63)	10 (59)		
	Never, divorced, or widowed	13 (39)	6 (37)	7 (41)		
**Employment status, n (%)**						.12
	Retired or unemployed	17 (52)	6 (37)	11 (65)		
	Employed	16 (48)	10 (63)	6 (35)		
**Relationship to the relative with dementia, n (%)**						.44
	Non-spousal family members	25 (76)	11 (69)	14 (82)		
	Spousal family members	8 (24)	5 (31)	3 (18)		
**Living with the relative with dementia, n (%)**						≥.99
	Living together	27 (82)	13 (81)	14 (82)		
	Not living together	6 (18)	3 (19)	3 (18)		
**Caregiving hours per week, n (%)**						.08
	40 hours or more	22 (67)	13 (81)	9 (53)		
	Less than 40 hours	11 (33)	3 (19)	8 (47)		
**Other family members helping with care, n (%)**						.85
	No	18 (55)	9 (56)	9 (53)		
	Yes	15 (45)	7 (44)	8 (47)		
**Experience in support groups, n (%)**						.88
	Never attended	19 (58)	9 (56)	10 (59)		
	Attending or attended before	14 (42)	7 (44)	7 (41)		
Years since the relative’s diagnosis of dementia, mean (SD; range)		4.8 (3.8; 0.4-14.2)	3.9 (2.9; 0.6-9.6)	5.6 (4.4; 0.4-14.2)	.19	
**Type of dementia in the relative, n (%)**						.56
	Alzheimer disease	13 (40)	5 (31)	8 (47)		
	Other dementias	11 (33)	7 (44)	4 (24)		
	Not sure	9 (27)	4 (25)	5 (29)		
**Stage of dementia in the relative, n (%)**						.89
	Early or mild	5 (15)	2 (13)	3 (18)		
	Middle or moderate	16 (49)	8 (50)	8 (47)		
	Late or severe	9 (27)	5 (31)	4 (23)		
	Not sure	3 (9)	1 (6)	2 (12)		

^a^ACT: acceptance and commitment therapy.

### Effects of the Videoconference-Delivered ACT Program on Outcomes

Multimedia Appendix 5 presents the overall *P* values for group, timepoint, and group×timepoint, along with their associated *F* values. The group×time interaction term was statistically significant for only the variable WHOQOL-BREF–Psych (*F*_2,52_=4.24; *P*=.020). All variables displayed statistically significant overall timepoint (within-group) effects (*F* values range from 3.32 to 20.73 and *P* values range from <.001 to .044), except for MM-CGI-BF (*F*_2,52_=2.60; *P*=.084).

Multimedia Appendix 6 presents the outcome measure scores in the pretest, posttest, and 3-month follow-up for each group, change scores in the posttest and 3-month follow-up from the pretest for each group, and findings from within-group and between-group comparisons in a single table. For a given variable in Multimedia Appendices 4 and 5, all inferential statistical results were obtained from the same linear mixed effects model. Within-group and between-group differences in outcomes in posttest and 3-month follow-up are presented in Table 2 and Table 3, respectively.

Two significant between-group differences were observed in the posttest. The ACT group reported significantly greater improvements than the control group in stress and psychological QoL in the posttest. For stress, the estimated within-group change in score for the ACT group (mean –7.75, SE 1.62 points; 95% CI –10.99 to –4.51; *P*<.001) compared to the control group (mean –3.03, SE 1.61 points; 95% CI –6.25 to 0.19; *P*=.065) resulted in an absolute between-group difference of mean –4.72 (SE 2.28) points (95% CI –9.29 to –0.15; *P*=.043). For psychological QoL, the estimated within-group change in score for the ACT group (mean 4.03, SE 0.96 points; 95% CI 2.10-5.96; *P*<.001) compared to the control group (mean 0.56, SE 0.96 points; 95% CI –1.37 to 2.48; *P*=.56) resulted in an absolute between-group difference of mean 3.48 (SE 1.36) points (95% CI 0.75-6.20; *P*=.014). No significant between-group differences were observed for other outcome measures in the posttest, and none were observed for any outcome measure in the 3-month follow-up (Tables 2 and 3).

For within-group comparisons in the ACT group, participants experienced a significant decrease in depressive symptoms, with a mean change of –6.09 (SE 1.16) points (95% CI –8.42 to –3.76; *P*<.001) in the posttest and –6.71 (SE 1.45) points (95% CI –9.63 to –3.81; *P*<.001) in the 3-month follow-up. These changes exceed the estimated MCID on the PHQ-9 scale [35]. In addition, the ACT group reported significant improvements in anxiety, stress, psychological QoL, caregiver burden, predeath grief, guilt, values-driven action, and experiential avoidance in both posttest and 3-month follow-up (Tables 2 and 3). For example, the ACT group experienced a significant decrease in anxiety symptoms, with a mean change of –4.41 (SE 1.14) points (95% CI –6.71 to –2.12; *P*<.001) at posttest and –4.09 (SE 1.47) points (95% CI –7.04 to –1.14; *P*=.008) in the 3-month follow-up. These changes exceed the estimated MCID on the GAD-7 [39,40]. No significant within-group differences in change scores for cognitive fusion and self-compassion in the ACT group were observed (Tables 2 and 3).

For within-group comparisons in the control group that received psychoeducation materials alone, participants experienced a significant decrease in depressive symptoms in the posttest and 3-month follow-up (Tables 2 and 3), but these changes were smaller than the estimated MCID on the PHQ-9 scale [35]. Additionally, the control group reported significant improvements in anxiety in 3-month follow-up, self-compassion at posttest, values-driven action in the posttest and 3-month follow-up, and experiential avoidance in the posttest (Tables 2 and 3).

**Table 2:** Within-group and between-group differences in outcomes at posttest in a pilot randomized controlled trial of a videoconference-delivered ACT^a^ group and a control group receiving psychoeducation materials for family caregivers with depression who give care to individuals with dementia in the United States.

	Estimated within-group Change Δ (posttest – pretest)^b^							Absolute between-group difference in Δ ACT versus Δ control at posttest^b^		
	ACT group			Control group			Mean (SE; 95% CI)			*P* value; ES
	Mean (SE; 95% CI)	*P* value; ES^c^	Mean (SE; 95% CI)		*P* value; ES					
PHQ-9^d^ (–)^e^	–6.09 (1.16; –8.42 to –3.76)	<.001; 1.31	–3.55 (1.15); (–5.87 to –1.24)		.003; 0.75	–2.54 (1.64; –5.82 to 0.75)			.13; 0.27	
GAD-7^f^ (–)^e^	–4.41 (1.14; –6.71 to –2.12)	<.001; 0.96	–2.14 (1.14; –4.42 to 0.15)		.067; 0.46	–2.27 (1.61; –5.51 to 0.97)			.17; 0.25	
PSS-10^g^ (–)^e^	–7.75 (1.62; –10.99 to –4.51)	<.001; 1.20	–3.03 (1.61; –6.25, 0.19)		.065; 0.46	–4.72 (2.28; –9.29 to –0.15)			.043; 0.36	
WHOQOL‑BREF–Psych^h^	4.03 (0.96; 2.10-5.96)	<.001; 1.05	0.56 (0.96; –1.37 to 2.48)		.56; 0.14	3.48 (1.36; 0.75-6.20)			.014; 0.45	
ZBI-12^i^ (–)^e^	–7.11 (1.92; –10.96 to –3.27)	<.001; 0.93	–2.34 (1.91; –6.16 to 1.49)		.23; 0.30	–4.78 (2.70; –10.21 to 0.65)			.083; 0.31	
MM-CGI-BF^j^ (–)^e^	–2.99 (1.45; –5.91 to –0.08)	.04; 0.51	–1.53 (1.44; –4.43 to 1.36)		.29; 0.26	–1.46 (2.05; –5.57 to 2.65)			.48; 0.12	
CGQ^k^ (–)^e^	–8.64 (3.45; –15.55 to –1.72)	.015; 0.63	–5.89 (3.43; –12.78 to 0.99)		.092; 0.42	–2.74 (4.86; –12.50 to 7.02)			.58; 0.10	
SCS-SF^l^	2.66 (2.01; –1.38 to 6.69)	.19; 0.33	5.79 (2.00; 1.79-9.80)		.005; 0.70	–3.14 (2.83; –8.82 to 2.54)			.27; 0.19	
ELS-9^m^	6.24 (1.42; 3.40-9.08)	<.001; 1.10	3.53 (1.41; 0.70-6.36)		.016; 0.61	2.71 (2.00; –1.30 to 6.72)			.18; 0.24	
AAQ-II^n^ (–)^e^	–5.16 (1.90; –8.97 to –1.34)	.009; 0.68	–4.14 (1.90; –7.95 to –0.34)		.033; 0.53	–1.01 (2.69; –6.40 to 4.38)			.71; 0.07	
CFQ-7^o^ (–)^e^	–1.29 (2.07; –5.44 to 2.86)	.54; 0.16	–0.28 (2.06; –4.42 to 3.86)		.89; 0.03	–1.01 (2.92; (–6.88 to 4.85)			.73; 0.06	

^a^ACT: Acceptance and commitment therapy.

^b^Established from linear mixed effects models.

^c^ES: effect size (Cohen *d*).

^d^PHQ-9: Patient Health Questionnaire-9.

^e^A minus sign in parentheses indicates that a decline in each variable means positive outcomes.

^f^GAD-7: Generalized Anxiety Disorder-7 Scale.

^g^PSS-10: Perceived Stress Scale -10.

^h^WHOQOL‑BREF-Psych: World Health Organization Quality of Life Assessment‑BREF-Psychological Health Component.

^i^ZBI-12: Zarit Burden Interview-12.

^j^MM-CGI-BF: Marwit–Meuser Caregiver Grief Inventory-Brief-Form.

^k^CGQ: Caregiver Guilt Questionnaire.

^l^SCS-SF: Self-Compassion Scale-Short Form.

^m^ELS-9: Engaged Living Scale-9.

^n^AAQ-II: Acceptance and Action Questionnaire-II.

^o^CFQ-7: Cognitive Fusion Questionnaire-7.

**Table 3:** Within-group and between-group differences in outcomes in 3-month follow-up in a pilot randomized controlled trial of a videoconference-delivered ACT^a^ group and a control group receiving psychoeducation materials for family caregivers with depression who give care to individuals with dementia in the United States.

	Estimated within-group change Δ (3-month follow-up – pretest)^b^							Absolute between-group difference in Δ ACT versus Δ control in 3-month follow-up^b^		
	ACT^a^ group			Control group			Mean (SE; 95% CI)			*P* value; ES
	Mean (SE; 95% CI)	*P* value; ES^c^	Mean (SE; 95% CI)		*P* value; ES					
PHQ-9^d^ (-)^e^	6.71 (1.45; –9.63 to –3.81)	<.001; 1.00	–4.89 (1.44; –7.78 to –2.00)		.001; 0.82	–1.83 (2.05; –5.93 to 2.28)			.38; 0.16	
GAD-7^f^ (–)^e^	–4.09 (1.47; –7.04 to –1.14)	.008; 0.69	–3.23 (1.46; –6.16 to –0.30)		.032; 0.54	–0.86 (2.07; –5.03 to 3.30)			.68; 0.07	
PSS-10^g^ (–)^e^	–7.04 (2.02; –11.09 to –2.99)	.001; 0.87	–1.95 (2.00; –5.96 to 2.07)		.34; 0.24	–5.10 (2.84; –10.80 to 0.60)			.079; 0.31	
WHOQOL‑BREF–Psych^h^	2.92 (1.19; 0.52-5.31)	.018; 0.61	2.16 (1.18; –0.21 to 4.53)		.073; 0.44	0.76 (1.68; –2.61 to 4.12)			.65; 0.08	
ZBI-12^i^ (–)^e^	–9.55 (2.47; –14.50 to –4.60)	<.001; 0.97	–3.60 (2.45; –8.51 to 1.31)		.15; 0.36	–5.95 (3.47; –12.92 to 1.02)			.093; 0.30	
MM-CGI-BF^j^ (–)^e^	–4.11 (1.82; –7.76 to –0.47)	.028; 0.57	–0.51 (1.80; –4.12 to 3.10)		.78; 0.07	–3.60 (2.56; –8.73 to 1.53)			.16; 0.25	
CGQ^k^ (–)^e^	–11.22 (4.39; –20.03 to –2.42)	.013; 0.64	–7.34 (4.35; –16.07 to 1.39)		.098; 0.41	–3.88 (6.18; –16.28 to 8.51)			.53; 0.11	
SCS-SF^l^	4.63 (2.48; –0.35 to 9.61)	.068; 0.47	3.55 (2.46; –1.38 to 8.48)		.15; 0.35	1.08 (3.49; –5.93 to 8.09)			.76; 0.05	
ELS-9^m^	5.33 (1.81; 1.69-8.97)	.005; 0.73	4.96 (1.80; 1.34-8.57)		.008; 0.67	0.37 (2.56; –4.76 to 5.50)			.89; 0.03	
AAQ-II^n^ (–)^e^	–6.48 (2.52; –11.55 to –1.42)	.013; 0.64	–3.32 (2.51; –8.35 to 1.72)		.19; 0.32	–3.17 (3.56; –10.31 to 3.98)			.38; 0.15	
CFQ-7^o^ (–)^e^	–5.10 (2.74; –10.59 to 0.39)	.068; 0.47	–3.59 (2.72; –9.05 to 1.87)		.19; 0.32	–1.51 (3.86; (–9.25 to 6.24)			.70; 0.07	

^a^ACT: acceptance and commitment therapy.

^a^Established from linear mixed effects models.

^c^ES: effect size (Cohen *d*).

^d^PHQ-9: Patient Health Questionnaire-9.

^e^A minus sign in parentheses indicates that a decline in each variable means positive outcomes.

^f^GAD-7: Generalized Anxiety Disorder-7 Scale.

^g^PSS-10: Perceived Stress Scale-10.

^h^WHOQOL‑BREF-Psych: World Health Organization Quality of Life Assessment‑BREF-Psychological Health Component.

^i^ZBI-12: Zarit Burden Interview-12.

^j^MM-CGI-BF: Marwit–Meuser Caregiver Grief Inventory-Brief-Form.

^k^CGQ: Caregiver Guilt Questionnaire.

^l^SCS-SF: Self-Compassion Scale-Short Form.

^m^ELS-9: Engaged Living Scale-9.

^n^AAQ-II: Acceptance and Action Questionnaire-II.

^o^CFQ-7: Cognitive Fusion Questionnaire-7.

## Discussion

### Principal Findings

This study, along with our previous work [18,19], represents the first efforts to evaluate videoconference-delivered ACT for family caregivers of individuals with ADRD. In particular, this pilot RCT targeted caregivers with at least mild depressive symptoms, differing from our earlier studies that included those with depressive, anxiety, or stress symptoms [18,19]. This decision was informed by previous findings that showed more positive outcomes when focusing specifically on caregivers with depressive symptoms [19], as well as meta-analyses indicating that web-based ACT interventions have larger effects on depressive symptoms when the population primarily has depressive symptoms [27].

Despite the small sample size, the videoconference-delivered ACT group demonstrated significantly greater improvements in stress and psychological QoL compared to the control group at posttest. The structured nature of ACT, delivered via videoconferencing with a coach, likely provided caregivers with opportunities to engage in real-time emotional processing and skill-building, enabling better responses to day-to-day stressful caregiving demands and situations [13,18]. This likely contributed to the greater improvements in stress and psychological QoL. Cohen *d* effect sizes for these between-group comparisons in stress and psychological QoL were 0.36 and 0.45, respectively, indicating small effect sizes. These findings align with meta-analyses on the effects of internet-delivered ACT compared to control conditions in RCTs across diverse populations [27], as well as the effects of ACT on family caregivers of individuals with chronic conditions [63]. Importantly, this is the first study to show significantly greater improvements in stress and psychological QoL for family caregivers of individuals with ADRD in an ACT program compared to a control group, providing valuable new evidence for this population.

However, no significant between-group differences were found for the other outcome measures in the posttest, nor for any outcome measure at the 3-month follow-up. This may be because this small-scale RCT was underpowered to detect between-group differences in most outcomes. Also, the control group received psychoeducation materials, which may have provided some benefits, particularly since more than half of the participants had not previously attended support groups. In addition, 1 participant in the control group, who found the study information through the clinical trial registration, had near-outlier data, which may have influenced some of the results. This participant’s data approached outlier status (ie, outside of 3 SDs from the mean). A sensitivity analysis was conducted excluding this participant’s data. The sensitivity analysis revealed statistically significant between-group differences in depressive symptoms in the posttest (absolute between-group difference=–3.25 points; *P*=.037); stress at posttest (absolute between-group difference=–6.45 points; *P*<.001) and in 3-month follow-up (absolute between-group difference=–7.65 points; *P*=.001); psychological QoL in the posttest (absolute between-group difference=4.35 points; *P*<.001); caregiver burden in the posttest (absolute between-group difference=–6.7 points; *P*=.003) and in the 3-month follow-up (absolute between-group difference=–8.83 points; *P*=.003); predeath grief in 3-month follow-up (absolute between-group difference=–5.31 points; *P*=.031); and values-driven action at posttest (absolute between-group difference=3.67 points; *P*=.032). The effect sizes for the differences between groups were larger in the posttest for all outcomes except for the SCS-SF and in the 3-month follow-up for all outcomes when excluding this participant’s data than when including it. Notably, the effect sizes shifted from small to medium in stress and caregiver burden in both the posttest and 3-month follow-up, as well as in psychological QoL in the posttest. A table detailing the results from this sensitivity analysis can be found in Multimedia Appendix 7.

For within-group comparisons, the videoconference-delivered ACT group experienced significant improvements in depressive symptoms, anxiety, stress, psychological QoL, caregiver burden, predeath grief, guilt, values-driven action, and experiential avoidance in both posttest and 3-month follow-up. The mean changes in the 3-month follow-up were similar to those in the posttest, indicating that the treatment effects were maintained over time. Additionally, Cohen *d* effect sizes for these within-group comparisons indicated moderate to large effect sizes in both posttest and 3-month follow-up for these outcomes. These findings align with within-group improvements observed in prior ACT studies delivered through various modes for this population [14-23]. While the control group, which received psychoeducational materials alone, showed some within-group improvements, these changes were smaller compared to the ACT group and were not statistically significant in most cases.

### Limitations

This study had several limitations. The small sample size may have limited the statistical power to detect small to moderate significant between-group differences in outcomes, especially at the 3-month follow-up. We adjusted our target sample size due to challenges in recruiting family caregivers with depression who give care to individuals with ADRD for an RCT within our funding period. Recruitment relied on sharing our study flyer with community sites, but we lacked access to the population through clinics, and some potential participants had already been recruited for previous studies, which further constrained our recruitment capacity. Another limitation of this pilot RCT is that, due to its exploratory and preliminary nature, no adjustment of *P* values was conducted for multiple statistical comparisons of the outcome measures. As a result, the findings should be interpreted with caution, and further studies with larger sample sizes and appropriate adjustments for multiple comparisons are needed to confirm the reliability and robustness of the results. This study did not collect data on participants’ income levels or the types of private caregiving support services they were receiving at the time of recruitment. Therefore, these factors were not considered in the data analysis when comparing group differences or controlling for these factors if between-group differences were identified. Given that financial resources and access to caregiving support services may influence caregivers’ mental health and engagement in interventions [64], future research should include these variables to better understand how socioeconomic status and caregiving resources impact mental health outcomes and intervention effects among family caregivers.

### Conclusions

The videoconference-delivered, therapist-guided ACT program demonstrated feasibility and positive effects on mental health outcomes among family caregivers with depression who give care to individuals with ADRD. Given the barriers many caregivers face in accessing in-person mental health services, this mode of delivery represents a promising alternative for improving caregivers’ mental health. Future studies are needed to replicate these findings with larger, more diverse caregiver populations and explore the long-term efficacy of videoconference-delivered ACT programs.

## Appendix

Multimedia Appendix 1 Overview of sessions provided to the videoconference-delivered acceptance and commitment therapy group for depressed family caregivers of individuals with dementia in the United States.

Multimedia Appendix 2 Participant demographic and caregiving-related questions administered to all participants at pretest in a pilot randomized controlled trial for depressed family caregivers of individuals with dementia in the United States.

Multimedia Appendix 3 CONSORT-EHEALTH (Consolidated Standards of Reporting Trials of Electronic and Mobile Health Applications and Online Telehealth) checklist.

Multimedia Appendix 4 Between-group differences in outcomes at pretest in a pilot randomized controlled trial of a videoconference-delivered acceptance and commitment therapy group versus a control group receiving psychoeducation materials for depressed family caregivers of individuals with dementia in the United States.

Multimedia Appendix 5 Results of overall between-group and within-group comparisons in a pilot randomized controlled trial of a videoconference-delivered acceptance and commitment therapy group versus a control group receiving psychoeducation materials for depressed family caregivers of individuals with dementia in the United States.

Multimedia Appendix 6 Scores in outcome measures with between-group and within-group comparisons in a pilot randomized controlled trial of a videoconference-delivered acceptance and commitment therapy group versus a control group receiving psychoeducation materials for depressed family caregivers of individuals with dementia in the United States.

Multimedia Appendix 7 Results of a sensitivity analysis excluding one participant with near-outlier data in a pilot randomized controlled trial of a videoconference-delivered acceptance and commitment therapy group versus a control group receiving psychoeducation materials for depressed family caregivers of individuals with dementia in the United States.

## Abbreviations

ACT: acceptance and commitment therapy
ADRD: Alzheimer's disease and related dementias
CGQ: Caregiver Guilt Questionnaire
CONSORT-EHEALTH: Consolidated Standards of Reporting Trials of Electronic and Mobile Health Applications and Online Telehealth
GAD-7: Generalized Anxiety Disorder-7 Scale
MCID: minimal clinically important difference
MM-CGI-BF: Marwit–Meuser Caregiver Grief Inventory-Brief-Form
PHQ-9: Patient Health Questionnaire-9
PSS-10: Perceived Stress Scale-10
QoL: quality of life
RCT: randomized controlled trial
SCS-SF: Self-Compassion Scale-Short Form
WHOQOL‑BREF-Psych: World Health Organization Quality of Life Assessment‑BREF-Psychological Health Component
ZBI-12: Zarit Burden Interview-12

## Data Availability

The findings from the data analysis are presented in the main document as Tables and Multimedia Appendices. Deidentified participant data are available from the corresponding author upon reasonable request.
