# Supplementary material for: Videoconference-Delivered Acceptance and Commitment Therapy for Family Caregivers of People With Dementia: Pilot Randomized Controlled Trial
Source: JMIR Form Res. 2025 Mar 31;9:e67545. doi: 10.2196/67545 (PMC11997529; doi:10.2196/67545)
Supplement: Multimedia Appendix 1 [file formative_v9i1e67545_app1.docx]

**Table S1.** Overview of sessions provided to the videoconference-delivered acceptance and commitment therapy group for depressed family caregivers of individuals with dementia in the United States.

| Sessions | Aims |
| --- | --- |
| Week 1. Orientation | - Orienting the caregiver to the intervention sessions and basic concepts of ACT.  - Conducting an initial interview to identify the caregiver’s difficulties in the six processes of ACT. |
| Week 2. Creative Hopelessness | - Helping the caregiver explore the efforts he or she has made to try to eliminate, change, or fix their difficult thoughts  - Guiding the caregiver in assessing the workability of these efforts. |
| Week 3. Control *vs.* Willingness | - Helping the caregiver acknowledge and consider experiencing uncomfortable and unwanted emotions and thoughts as they are, as an alternative to control.  - Guiding the caregiver in exploring barriers to willingness and practicing exercises to overcome those barriers. |
| Week 4. Acceptance | - Fostering the caregiver’s willingness/acceptance using case scenario exercises.  - Coaching the caregiver in learning and applying strategies adapted to their patterns of experiential avoidance. |
| Week 5.Defusion & Observing self | - Coaching the caregiver in learning and applying exercises to step back or detach from unhelpful thoughts and emotions, and to observe thoughts and emotions without judgment. |
| Week 6. Observing self & Being present | - Coaching the caregiver in practicing exercises to observe thoughts and emotions without judgment.  - Coaching the caregiver in learning and practicing mindfulness exercises to promote maintaining contact with the present moment. |
| Week 7. Values | - Assisting the caregiver in clarifying the core values that give meaning/purpose to the caregiving role and other non-care-related values in his/her life.  - Assisting the caregiver in identifying potential barriers to living a values-focused life and ways to overcome these barriers. |
| Week 8. Committed action | - Assisting the caregiver in setting a committed action plan.  - Orienting the caregiver to the use of activity scheduling and monitoring for involvement in committed actions during the week. |
| Week 9. Committed action | - Reviewing the caregiver’s performance of committed actions and mood in the past week, discussing barriers that hindered committed actions, and identifying ways to overcome those barriers.  - Revising the action plan and activity scheduling for the following week as needed.  - Coaching the caregiver in the use of mindfulness and defusion skills to follow through on committed actions in the face of painful thoughts and feelings. |
| Week 10. Closure | - Wrapping up and assisting the caregiver in revising the committed action plan for values-based living.  *Caregivers are provided with additional worksheets and online ACT resources for continued use until the booster session. |
| A booster session | - Discussing how the caregiver has been doing since the end of weekly sessions.  - Coaching the caregiver in necessary ACT skill practices and setting action plans. |
