# Supplementary material for: Videoconference-Delivered Acceptance and Commitment Therapy for Family Caregivers of People With Dementia: Pilot Randomized Controlled Trial
Source: JMIR Form Res. 2025 Mar 31;9:e67545. doi: 10.2196/67545 (PMC11997529; doi:10.2196/67545)
Supplement: Multimedia Appendix 2 [file formative_v9i1e67545_app2.docx]

**Table S2.** Participant demographic and caregiving-related questions administered to all participants at pretest in a pilot randomized controlled trial for depressed family caregivers of individuals with dementia in the United States.

| Age | _________________ years |
| --- | --- |
| Gender | [1] Male [2] Female |
| Level of Education | [1] Less than high school [2] High school graduate  [3] Technical, trade, or vocational school [4] Some college  [5] Bachelor’s degree completed [6] Postgraduate |
| Race | [1] Non-Hispanic White [2] Black or African American  [3] Hispanic or Latino [4] Native American or American Indian  [5] Asian [6] Other (Please specify: ) |
| Marital Status | [1] Married/ Living with a partner [2] Divorced/ Separated  [3] Widowed [4] Single/ Never married |
| Employment Status | [1] Employed full time [2] Employed part time  [3] Unemployed [4] Retired |
| Relation to the relative with dementia (e.g., If you are a wife, you check wife) | [1] Wife [2] Husband [3] Daughter  [4] Son [5] Sister [6] Daughter-in-law  [7] Son-in-law [8] Other: _____________ |
| Living with the relative with dementia | [1] Living together [2] Not living together |
| Caregiving hours per week | [1] 40 hours or over [2] 21-39 hours  [3] 9-20 hours [4] 8 hours or less |
| Other family members who help with the care | [1] No  [2] Yes (who and how much? ________________________________) |
| Experiences in support groups for caregivers of people with dementia | [1] I currently attend a support group for dementia caregivers.  If so, how often do you attend the support group? ________________  [2] I attended a support group before, but I am not going anymore.  [3] I have never attended a support group. |
| Diagnosis of your relative’s dementia | Year: ___________________ Month: _____________________ |
| Type of your relative’s dementia | [1] Alzheimer’s disease [2] Vascular dementia  [3] Frontotemporal dementia [4] Dementia with Lewy Bodies  [5] Other: ______________ [6] Not sure |
| Stage of your relative’s dementia | [1] Early stage/ Mild [2] Middle stage/ Moderate  [3] Late stage/ Severe [4] Not sure |
