# Supplementary material for: Videoconference-Delivered Acceptance and Commitment Therapy for Family Caregivers of People With Dementia: Pilot Randomized Controlled Trial
Source: JMIR Form Res. 2025 Mar 31;9:e67545. doi: 10.2196/67545 (PMC11997529; doi:10.2196/67545)
Supplement: Multimedia Appendix 4 [file formative_v9i1e67545_app4.docx]

**Table S3.** Between-group differences in outcomes at pretest in a pilot randomized controlled trial of a videoconference-delivered acceptance and commitment therapy group versus a control group receiving psychoeducation materials for depressed family caregivers of individuals with dementia in the United States.

| Variables | | t-test for Equality of Means | | | | | | | |
| --- | --- | --- | --- | --- | --- | --- | --- | --- | --- |
|  |  | t | df | Significance | | Mean Difference | Std. Dev. Difference | 95% Confidence Interval of the Difference | |
|  |  |  |  | Two-Sided P value | |  |  | Lower | Upper |
| PHQ-9 |  | 0.06 | 31 | 0.949 |  | 0.11 | 5.11 | -3.52 | 3.74 |
| GAD-7 |  | -0.56 | 31 | 0.577 |  | -1.07 | 5.45 | -4.94 | 2.80 |
| PSS-10 |  | 0.21 | 31 | 0.834 |  | 0.49 | 6.62 | -4.22 | 5.19 |
| WHOQOL‑BREF- Psych |  | -0.63 | 31 | 0.534 |  | -0.84 | 3.82 | -3.55 | 1.88 |
| ZBI-12 |  | 1.60 | 31 | 0.120 |  | 5.08 | 9.12 | -1.40 | 11.56 |
| MM-CGI-BF |  | -0.96 | 31 | 0.343 |  | -1.91 | 5.70 | -5.96 | 2.14 |
| CGQ |  | 0.56 | 31 | 0.581 |  | 2.84 | 14.62 | -7.54 | 13.22 |
| SCS-SF |  | 0.53 | 31 | 0.599 |  | 1.49 | 8.04 | -4.22 | 7.20 |
| ELS-9 |  | -0.11 | 31 | 0.914 |  | -0.26 | 7.06 | -5.28 | 4.74 |
| AAQ-II |  | 0.05 | 31 | 0.961 |  | 0.19 | 10.82 | -7.50 | 7.87 |
| CFQ-7 |  | -0.02 | 31 | 0.988 |  | -0.06 | 11.47 | -8.21 | 8.08 |

Abbreviations: AAQ-II, Acceptance and Action Questionnaire-II; CGQ, Caregiver Guilt Questionnaire; CFQ-7, Cognitive Fusion Questionnaire-7; ELS-9, Engaged Living Scale -9; GAD-7, Generalized Anxiety Disorder-7; MM-CGI-BF, Marwit–Meuser Caregiver Grief Inventory-Brief-Form; PHQ-9, Patient Health Questionnaire-9; PSS-10, Perceived Stress Scale -10; SCS-SF, Self-Compassion Scale-Short Form; WHOQOL‑BREF-Psych, World Health Organization Quality of Life Assessment‑BREF-Psychological Health Component; ZBI-12, Zarit Burden Interview-12.
