# Supplementary material for: Videoconference-Delivered Acceptance and Commitment Therapy for Family Caregivers of People With Dementia: Pilot Randomized Controlled Trial
Source: JMIR Form Res. 2025 Mar 31;9:e67545. doi: 10.2196/67545 (PMC11997529; doi:10.2196/67545)
Supplement: Multimedia Appendix 5 [file formative_v9i1e67545_app5.docx]

**Table S4.** Results of overall between-group and within-group comparisons in a pilot randomized controlled trial of a videoconference-delivered acceptance and commitment therapy group versus a control group receiving psychoeducation materials for depressed family caregivers of individuals with dementia in the United States.

|  | ACT Group (n=16) | | | Control Group with Psychoeducation Materials Only (n=17) | | | Overall Significance (F value, p-value) | | |
| --- | --- | --- | --- | --- | --- | --- | --- | --- | --- |
|  | Pretest  Mean ± SD  (SE) | Posttest  Mean ± SD  (SE) | 3-mo F/U  Mean ± SD  (SE) | Pretest  Mean ± SD  (SE) | Posttest  Mean ± SD  (SE) | 3-mo F/U  Mean ± SD  (SE) | Group^a^  (Between-Group) | Timepoint^a^  (Within-Group) | Group x Timepoint  Interaction^a^ |
| **Variables** | | | | | | | | | |
| PHQ-9 (-)^b^ | 11.94 ± 4.54  (1.13) | 5.71 ± 3.91  (1.05) | 5.14 ± 5.16  (1.38) | 11.82 ± 5.59  (1.36) | 8.50 ± 4.26  (1.14) | 7.07 ± 5.20  (1.39) | F = 0.86  p = 0.360 | F = 20.73  p < 0.001 | F = 1.20  p = 0.308 |
| GAD-7 (-)^b^ | 8.81 ± 4.45  (1.11) | 4.29 ± 2.81  (.75) | 4.64 ± 5.20  (1.40) | 9.88 ± 6.25  (1.52) | 8.36 ± 5.65  (1.51) | 7.07 ± 6.17  (1.65) | F = 1.63  p = 0.212 | F = 8.98  p < 0.001 | F = 1.19  p = 0.314 |
| PSS-10 (-)^b^ | 23.31 ± 4.48  (1.12) | 15.71 ± 4.41  (1.18) | 16.36 ± 5.37  (1.44) | 22.82 ± 8.13  (1.97) | 20.86 ± 6.59  (1.76) | 21.50 ± 8.86  (2.37) | F = 1.98  p = 0.170 | F = 11.24  p < 0.001 | F = 2.36  p = 0.104 |
| WHOQOL  ‑BREF  - Psych | 16.75 ± 3.21  (.80) | 21.00 ± 3.68  (.98) | 19.79 ± 4.15  (1.11) | 17.59 ± 4.32  (1.05) | 18.21 ± 3.36  (.90) | 19.79 ± 4.28  (1.14) | F = 0.26  p = 0.615 | F = 6.45  p = 0.003 | F = 4.24  p = 0.020 |
| ZBI-12 (-)^b^ | 31.38 ± 6.20  (1.55) | 23.57 ± 6.72  (1.80) | 21.36 ± 7.80  (2.09) | 26.29 ±11.18  (2.71) | 25.36 ±11.08  (2.96) | 23.64 ± 9.28  (2.48) | F = 0.29  p = 0.591 | F = 8.15  p < 0.001 | F = 1.85  p = 0.167 |
| MM-CGI-BF (-)^b^ | 19.50 ± 5.83  (1.46) | 16.21 ± 5.10  (1.36) | 15.21 ± 5.92  (1.58) | 21.41 ± 5.57  (1.35) | 19.93 ± 6.27  (1.68) | 20.93 ± 7.45  (1.99) | F = 4.07  p = 0.052 | F = 2.60  p = 0.084 | F = 1.01  p = 0.371 |
| CGQ (-)^b^ | 42.31 ±11.67  (2.92) | 33.43 ±16.57  (4.43) | 30.93 ±17.00  (4.54) | 39.47 ±16.92  (4.10) | 34.93 ±17.87  (4.78) | 33.00 ± 13.62  (3.64) | F = 0.02  p = 0.893 | F = 5.51p = 0.007 | F = 0.22  p = 0.802 |
| SCS-SF | 34.31 ± 6.31  (1.58) | 37.07 ± 8.07  (2.16) | 39.00 ± 6.86  (1.83) | 32.82 ± 9.38  (2.27) | 38.71 ± 9.08  (2.43) | 36.43 ± 8.66  (2.32) | F = 0.12  p = 0.733 | F = 4.67  p = 0.014 | F = 1.37  p = 0.262 |
| ELS-9 | 26.50 ± 5.74  (1.43) | 33.21 ± 5.62  (1.50) | 32.14 ± 5.39  (1.44) | 26.76 ± 8.10  (1.96) | 30.07 ± 5.72  (1.53) | 31.57 ± 7.13  (1.91) | F = 0.15  p = 0.706 | F = 12.66  p < 0.001 | F = 1.35  p = 0.269 |
| AAQ-II (-)^b^ | 26.19 ± 9.05  (2.26) | 20.50 ± 8.34  (2.23) | 19.29 ±10.64  (2.84) | 26.00 ±12.24  (2.97) | 22.36 ±10.12  (2.70) | 23.07 ± 12.29  (3.29) | F = 0.12  p = 0.730 | F = 6.19  p = 0.004 | F = 0.44  p = 0.649 |
| CFQ-7 (-)^b^ | 25.94 ±10.04  (2.51) | 23.93 ± 11.11  (2.97) | 20.29 ± 10.67  (2.85) | 26.00 ± 12.66  (3.07) | 26.21 ±12.06  (3.22) | 22.79 ± 11.87  (3.17) | F = 0.06  p = 0.807 | F = 3.32  p = 0.044 | F = 0.08  p = 0.920 |
|  | | | | | | | | | |
| ^a^ Established from linear mixed effects models.  ^b^ A minus sign in parentheses indicates that a decline in each variable means positive outcomes.  Abbreviations: AAQ-II, Acceptance and Action Questionnaire-II; ACT, acceptance and commitment therapy; CGQ, Caregiver Guilt Questionnaire; CFQ-7, Cognitive Fusion Questionnaire-7; ELS-9, Engaged Living Scale -9; ES, effect size; F/U, follow-up; GAD-7, Generalized Anxiety Disorder-7; MM-CGI-BF, Marwit–Meuser Caregiver Grief Inventory-Brief-Form; PHQ-9, Patient Health Questionnaire-9; PSS-10, Perceived Stress Scale -10; SCS-SF, Self-Compassion Scale-Short Form; SD, standard deviation; SE, standard error; WHOQOL‑BREF-Psych, World Health Organization Quality of Life Assessment‑BREF-Psychological Health Component; ZBI-12, Zarit Burden Interview-12. | | | | | | | | | |
