# Supplementary material for: Videoconference-Delivered Acceptance and Commitment Therapy for Family Caregivers of People With Dementia: Pilot Randomized Controlled Trial
Source: JMIR Form Res. 2025 Mar 31;9:e67545. doi: 10.2196/67545 (PMC11997529; doi:10.2196/67545)
Supplement: Multimedia Appendix 6 [file formative_v9i1e67545_app6.docx]

**Table S5.** Scores in outcome measures with between-group and within-group comparisons in a pilot randomized controlled trial of a videoconference-delivered acceptance and commitment therapy group versus a control group receiving psychoeducation materials for depressed family caregivers of individuals with dementia in the United States.

|  | ACT Group (n=16) | | | Control Group with Psychoeducation Materials Only (n=17) | | | Estimated Within-Group Change  Δ (Posttest – Pretest)^a^ | | Absolute Between-Group  Difference in Δ ACT vs.  Δ Control^a^  at Posttest  Mean (SE)  (95% CI)  p-value  ES | Estimated Within-Group Change  Δ (3-mo FU – Pretest) ^a^ | | Absolute  Between-Group  Difference  in Δ ACT vs.  Δ Control^a^  at 3-mo FU  Mean (SE)  (95% CI)  p-value  ES |
| --- | --- | --- | --- | --- | --- | --- | --- | --- | --- | --- | --- | --- |
|  | Pretest  Mean ± SD  (SE) | Posttest  Mean ± SD  (SE) | 3-mo F/U  Mean ± SD  (SE) | Pretest  Mean ± SD  (SE) | Posttest  Mean ± SD  (SE) | 3-mo F/U  Mean ± SD  (SE) | ACT  Mean (SE)  (95% CI)  p-value  ES | Control  Mean (SE)  (95% CI)  p-value  ES |  | ACT  Mean (SE)  (95% CI)  p-value  ES | Control  Mean (SE)  (95% CI)  p-value  ES |  |
| **Variables** | | | | | | | | | | | | |
| PHQ-9 (-)^b^ | 11.94 ± 4.54  (1.13) | 5.71 ± 3.91  (1.05) | 5.14 ± 5.16  (1.38) | 11.82 ± 5.59  (1.36) | 8.50 ± 4.26  (1.14) | 7.07 ± 5.20  (1.39) | -6.09 (1.16)  (-8.42, -3.76)  <0.001  1.31 | -3.55 (1.15)  (-5.87, -1.24)  0.003  0.75 | -2.54 (1.64)  (-5.82, 0.75)  0.13  0.27 | -6.71 (1.45)  (-9.63, -3.81)  <0.001  1.00 | -4.89 (1.44)  (-7.78, -2.00)  0.001  0.82 | -1.83 (2.05)  (-5.93, 2.28)  0.38  0.16 |
| GAD-7 (-)^b^ | 8.81 ± 4.45  (1.11) | 4.29 ± 2.81  (.75) | 4.64 ± 5.20  (1.40) | 9.88 ± 6.25  (1.52) | 8.36 ± 5.65  (1.51) | 7.07 ± 6.17  (1.65) | -4.41 (1.14)  (-6.71, -2.12)  <0.001  0.96 | -2.14 (1.14)  (-4.42, 0.15)  0.067  0.46 | -2.27 (1.61)  (-5.51, 0.97)  0.17  0.25 | -4.09 (1.47)  (-7.04, -1.14)  0.008  0.69 | -3.23 (1.46)  (-6.16, -0.30)  0.032  0.54 | -0.86 (2.07)  (-5.03, 3.30)  0.68  0.07 |
| PSS-10 (-)^b^ | 23.31 ± 4.48  (1.12) | 15.71 ± 4.41  (1.18) | 16.36 ± 5.37  (1.44) | 22.82 ± 8.13  (1.97) | 20.86 ± 6.59  (1.76) | 21.50 ± 8.86  (2.37) | -7.75 (1.62)  (-10.99, -4.51)  <0.001  1.20 | -3.03 (1.61)  (-6.25, 0.19)  0.065  0.46 | -4.72 (2.28)  (-9.29, -0.15)  0.043  0.36 | -7.04 (2.02)  (-11.09, -2.99)  0.001  0.87 | -1.95 (2.00)  (-5.96, 2.07)  0.34  0.24 | -5.10 (2.84)  (-10.80, 0.60)  0.079  0.31 |
| WHOQOL  ‑BREF  - Psych | 16.75 ± 3.21  (.80) | 21.00 ± 3.68  (.98) | 19.79 ± 4.15  (1.11) | 17.59 ± 4.32  (1.05) | 18.21 ± 3.36  (.90) | 19.79 ± 4.28  (1.14) | 4.03 (0.96)  (2.10, 5.96)  <0.001  1.05 | 0.56 (0.96)  (-1.37, 2.48)  0.56  0.14 | 3.48 (1.36)  (0.75, 6.20)  0.014  0.45 | 2.92 (1.19)  (0.52, 5.31)  0.018  0.61 | 2.16 (1.18)  (-0.21, 4.53)  0.073  0.44 | 0.76 (1.68)  (-2.61, 4.12)  0.65  0.08 |
| ZBI-12 (-)^b^ | 31.38 ± 6.20  (1.55) | 23.57 ± 6.72  (1.80) | 21.36 ± 7.80  (2.09) | 26.29 ± 11.18  (2.71) | 25.36 ± 11.08  (2.96) | 23.64 ± 9.28  (2.48) | -7.11 (1.92)  (-10.96, -3.27)  <0.001  0.93 | -2.34 (1.91)  (-6.16, 1.49)  0.23  0.30 | -4.78 (2.70)  (-10.21, 0.65)  0.083  0.31 | -9.55 (2.47)  (-14.50, -4.60)  <0.001  0.97 | -3.60 (2.45)  (-8.51, 1.31)  0.15  0.36 | -5.95 (3.47)  (-12.92, 1.02)  0.093  0.30 |
| MM-CGI-BF (-)^b^ | 19.50 ± 5.83  (1.46) | 16.21 ± 5.10  (1.36) | 15.21 ± 5.92  (1.58) | 21.41 ± 5.57  (1.35) | 19.93 ± 6.27  (1.68) | 20.93 ± 7.45  (1.99) | -2.99 (1.45)  (-5.91, -0.08)  0.04  0.51 | -1.53 (1.44)  (-4.43, 1.36)  0.29  0.26 | -1.46 (2.05)  (-5.57, 2.65)  0.48  0.12 | -4.11 (1.82)  (-7.76, -0.47)  0.028  0.57 | -0.51 (1.80)  (-4.12, 3.10)  0.78  0.07 | -3.60 (2.56)  (-8.73, 1.53)  0.16  0.25 |
| CGQ (-)^b^ | 42.31 ± 11.67  (2.92) | 33.43 ± 16.57  (4.43) | 30.93 ± 17.00  (4.54) | 39.47 ± 16.92  (4.10) | 34.93 ± 17.87  (4.78) | 33.00 ± 13.62  (3.64) | -8.64 (3.45)  (-15.55, -1.72)  0.015  0.63 | -5.89 (3.43)  (-12.78, 0.99)  0.092  0.42 | -2.74 (4.86)  (-12.50, 7.02)  0.58  0.10 | -11.22 (4.39)  (-20.03, -2.42)  0.013  0.64 | -7.34 (4.35)  (-16.07, 1.39)  0.098  0.41 | -3.88 (6.18)  (-16.28, 8.51)  0.53  0.11 |
| SCS-SF | 34.31 ± 6.31  (1.58) | 37.07 ± 8.07  (2.16) | 39.00 ± 6.86  (1.83) | 32.82 ± 9.38  (2.27) | 38.71 ± 9.08  (2.43) | 36.43 ± 8.66  (2.32) | 2.66 (2.01)  (-1.38, 6.69)  0.19  0.33 | 5.79 (2.00)  (1.79, 9.80)  0.005  0.70 | -3.14 (2.83)  (-8.82, 2.54)  0.27  0.19 | 4.63 (2.48)  (-0.35, 9.61)  0.068  0.47 | 3.55 (2.46)  (-1.38, 8.48)  0.15  0.35 | 1.08 (3.49)  (-5.93, 8.09)  0.76  0.05 |
| ELS-9 | 26.50 ± 5.74  (1.43) | 33.21 ± 5.62  (1.50) | 32.14 ± 5.39  (1.44) | 26.76 ± 8.10  (1.96) | 30.07 ± 5.72  (1.53) | 31.57 ± 7.13  (1.91) | 6.24 (1.42)  (3.40, 9.08)  <0.001  1.10 | 3.53 (1.41)  (0.70, 6.36)  0.016  0.61 | 2.71 (2.00)  (-1.30, 6.72)  0.18  0.24 | 5.33 (1.81)  (1.69, 8.97)  0.005  0.73 | 4.96 (1.80)  (1.34, 8.57)  0.008  0.67 | 0.37 (2.56)  (-4.76, 5.50)  0.89  0.03 |
| AAQ-II (-)^b^ | 26.19 ± 9.05  (2.26) | 20.50 ± 8.34  (2.23) | 19.29 ± 10.64  (2.84) | 26.00 ± 12.24  (2.97) | 22.36 ± 10.12  (2.70) | 23.07 ± 12.29  (3.29) | -5.16 (1.90)  (-8.97, -1.34)  0.009  0.68 | -4.14 (1.90)  (-7.95, -0.34)  0.033  0.53 | -1.01 (2.69)  (-6.40, 4.38)  0.71  0.07 | -6.48 (2.52)  (-11.55, -1.42)  0.013  0.64 | -3.32 (2.51)  (-8.35, 1.72)  0.19  0.32 | -3.17 (3.56)  (-10.31, 3.98)  0.38  0.15 |
| CFQ-7 (-)^b^ | 25.94 ± 10.04  (2.51) | 23.93 ± 11.11  (2.97) | 20.29 ± 10.67  (2.85) | 26.00 ± 12.66  (3.07) | 26.21 ± 12.06  (3.22) | 22.79 ± 11.87  (3.17) | -1.29 (2.07)  (-5.44, 2.86)  0.54  0.16 | -0.28 (2.06)  (-4.42, 3.86)  0.89  0.03 | -1.01 (2.92)  (-6.88, 4.85)  0.73  0.06 | -5.10 (2.74)  (-10.59, 0.39)  0.068  0.47 | -3.59 (2.72)  (-9.05, 1.87)  0.19  0.32 | -1.51 (3.86)  (-9.25, 6.24)  0.70  0.07 |
|  | | | | | | | | | | | | |
| ^a^ Established from linear mixed effects models.  ^b^ A minus sign in parentheses indicates that a decline in each variable means positive outcomes.  Abbreviations: AAQ-II, Acceptance and Action Questionnaire-II; ACT, acceptance and commitment therapy; CGQ, Caregiver Guilt Questionnaire; CFQ-7, Cognitive Fusion Questionnaire-7; ELS-9, Engaged Living Scale -9; ES, effect size; F/U, follow-up; GAD-7, Generalized Anxiety Disorder-7; MM-CGI-BF, Marwit–Meuser Caregiver Grief Inventory-Brief-Form; PHQ-9, Patient Health Questionnaire-9; PSS-10, Perceived Stress Scale -10; SCS-SF, Self-Compassion Scale-Short Form; SD, standard deviation; SE, standard error; WHOQOL‑BREF-Psych, World Health Organization Quality of Life Assessment‑BREF-Psychological Health Component; ZBI-12, Zarit Burden Interview-12. | | | | | | | | | | | | |
