# Supplementary material for: Videoconference-Delivered Acceptance and Commitment Therapy for Family Caregivers of People With Dementia: Pilot Randomized Controlled Trial
Source: JMIR Form Res. 2025 Mar 31;9:e67545. doi: 10.2196/67545 (PMC11997529; doi:10.2196/67545)
Supplement: Multimedia Appendix 7 [file formative_v9i1e67545_app7.docx]

**Table S6.** Results of a sensitivity analysis excluding one participant with near-outlier data in a pilot randomized controlled trial of a videoconference-delivered acceptance and commitment therapy group versus a control group receiving psychoeducation materials for depressed family caregivers of individuals with dementia in the United States.

| Variable |  | Posttest - Pretest for ACT | Posttest - Pretest for Control | Δ ACT vs. Δ Control at Posttest |  | 3-mo F/U - Pretest for ACT | 3-mo F/U - Pretest for Control | Δ ACT vs. Δ Control at 3-mo F/U |
| --- | --- | --- | --- | --- | --- | --- | --- | --- |
| **PHQ-9 (-)** |  |  |  |  |  |  |  |  |
| Mean change (smaller or greater than the original data?) |  | -6.08 | -2.83 (smaller change) | -3.25 (greater change) |  | -6.71 | -3.54 (smaller change) | -3.16 (greater change) |
| P value |  | <0.001 | 0.012 | 0.037 |  | <0.001 | 0.013 | 0.11 |
| Interpretation in terms of statistical significance |  | Same | Same | **Different (changed from non-significant to significant)** |  | Same | Same | Same |
| Effect size (for between-group differences) |  |  |  | 0.38 (changed from 0.27) |  |  |  | 0.29 (changed from 0.16) |
| **GAD-7 (-)** |  |  |  |  |  |  |  |  |
| Mean change (smaller or greater than the original data?) |  | -4.41 | -1.53 (smaller change) | -2.87 (greater change) |  | -4.08 | -1.98 (smaller change) | -2.1 (greater change) |
| P value |  | <0.001 | 0.17 | 0.065 |  | 0.005 | 0.17 | 0.29 |
| Interpretation in terms of statistical significance |  | Same | Same | Same |  | Same | **Different (changed from significant to non-significant)** | Same |
| Effect size (for between-group differences) |  |  |  | 0.33 (changed from 0.25) |  |  |  | 0.19 (changed from 0.07) |
| **PSS-10 (-)** |  |  |  |  |  |  |  |  |
| Mean change (smaller or greater than the original data?) |  | -7.78 | -1.33 (smaller change) | -6.45 (greater change) |  | -7.08 | 0.57 (changed direction; original = -1.95) | -7.65 (greater change) |
| P value |  | <0.001 | 0.3 | <0.001 |  | <0.001 | 0.73 | 0.001 |
| Interpretation in terms of statistical significance |  | Same | Same | Same |  | Same | Same | **Different (changed from non-significant to significant)** |
| Effect size (for between-group differences) |  |  |  | **0.65, medium effect size (changed from 0.36, small effect size)** |  |  |  | **0.60, medium effect size (changed from 0.31, small effect size)** |
| **WHOQOL‑BREF-Psych** |  |  |  |  |  |  |  |  |
| Mean change (smaller or greater than the original data?) |  | 3.99 | -0.36 (changed direction; original = 0.56) | 4.35 (greater change) |  | 2.87 | 0.56 (smaller change) | 2.31 (greater change) |
| P value |  | <0.001 | 0.65 | <0.001 |  | 0.005 | 0.58 | 0.11 |
| Interpretation in terms of statistical significance |  | Same | Same | Same |  | Same | Same | Same |
| Effect size (for between-group differences) |  |  |  | **0.70, medium effect size (changed from 0.45, small effect size)** |  |  |  | 0.29 (changed from 0.08) |
| **ZBI-12 (-)** |  |  |  |  |  |  |  |  |
| Mean change (smaller or greater than the original data?) |  | -7.02 | -0.32 (smaller change) | -6.7 (greater change) |  | -9.42 | -0.59 (smaller change) | -8.83 (greater change) |
| P value |  | <0.001 | 0.84 | 0.003 |  | <0.001 | 0.77 | 0.003 |
| Interpretation in terms of statistical significance |  | Same | Same | **Different (changed from non-significant to significant)** |  | Same | Same | **Different (changed from non-significant to significant)** |
| Effect size (for between-group differences) |  |  |  | **0.55, medium effect size (changed from 0.31, small effect size)** |  |  |  | **0.55, medium effect size (changed from 0.30, small effect size)** |
| **MM-CGI-BF (-)** |  |  |  |  |  |  |  |  |
| Mean change (smaller or greater than the original data?) |  | -2.97 | -0.4 (smaller change) | -2.58 (greater change) |  | -4.09 | 1.21 (changed direction; original = -0.51) | -5.31 (greater change) |
| P value |  | 0.028 | 0.77 | 0.18 |  | 0.018 | 0.48 | 0.031 |
| Interpretation in terms of statistical significance |  | Same | Same | Same |  | Same | Same | **Different (changed from non-significant to significant)** |
| Effect size (for between-group differences) |  |  |  | 0.24 (changed from 0.12) |  |  |  | 0.39 (changed from 0.25) |
| **CGQ (-)** |  |  |  |  |  |  |  |  |
| Mean change (smaller or greater than the original data?) |  | -8.61 | -2.52 (smaller change) | -6.09 (greater change) |  | -11.2 | -3.36 (smaller change) | -7.83 (greater change) |
| P value |  | 0.006 | 0.42 | 0.17 |  | 0.006 | 0.41 | 0.17 |
| Interpretation in terms of statistical significance |  | Same | Same | Same |  | Same | Same | Same |
| Effect size (for between-group differences) |  |  |  | 0.24 (changed from 0.10) |  |  |  | 0.24 (changed from 0.11) |
| **SCS-SF** |  |  |  |  |  |  |  |  |
| Mean change (smaller or greater than the original data?) |  | 2.63 | 3.08 (smaller change) | -0.45 (smaller change) |  | 4.61 | 0.26 (smaller change) | 4.34 (greater change) |
| P value |  | 0.082 | 0.05 | 0.83 |  | 0.02 | 0.89 | 0.12 |
| Interpretation in terms of statistical significance |  | Same | Same | Same |  | Same | Same | Same |
| Effect size (for between-group differences) |  |  |  | 0.04 (changed from 0.19) |  |  |  | 0.28 (changed from 0.05) |
| **ELS-9** |  |  |  |  |  |  |  |  |
| Mean change (smaller or greater than the original data?) |  | 6.18 | 2.51 (smaller change) | 3.67 (greater change) |  | 5.25 | 2.72 (smaller change) | 2.53 (greater change) |
| P value |  | <0.001 | 0.041 | 0.032 |  | 0.001 | 0.09 | 0.25 |
| Interpretation in terms of statistical significance |  | Same | Same | **Different (changed from non-significant to significant)** |  | Same | **Different (changed from significant to non-significant)** | Same |
| Effect size (for between-group differences) |  |  |  | 0.39 (changed from 0.24) |  |  |  | 0.20 (changed from 0.03) |
| **AAQ-II (-)** |  |  |  |  |  |  |  |  |
| Mean change (smaller or greater than the original data?) |  | -5.12 | -2.63 (smaller change) | -2.49 (greater change) |  | -6.43 | -0.5 (smaller change) | -5.93 (greater change) |
| P value |  | 0.002 | 0.12 | 0.29 |  | 0.005 | 0.82 | 0.064 |
| Interpretation in terms of statistical significance |  | Same | **Different (changed from significant to non-significant)** | Same |  | Same | Same | Same |
| Effect size (for between-group differences) |  |  |  | 0.19 (changed from 0.07) |  |  |  | 0.33 (changed from 0.15) |
| **CFQ-7 (-)** |  |  |  |  |  |  |  |  |
| Mean change (smaller or greater than the original data?) |  | -1.25 | 0.16 (changed direction; original = -0.28) | -1.41 (greater change) |  | -5.04 | -1.23 (smaller change) | -3.81 (greater change) |
| P value |  | 0.5 | 0.93 | 0.6 |  | 0.047 | 0.63 | 0.29 |
| Interpretation in terms of statistical significance |  | Same | Same | Same |  | Same | Same | Same |
| Effect size (for between-group differences) |  |  |  | 0.09 (changed from 0.06) |  |  |  | 0.19 (changed from 0.07) |

Note: A minus sign in parentheses indicates that a decline in each variable means positive outcomes.

Abbreviations: AAQ-II, Acceptance and Action Questionnaire-II; ACT, acceptance and commitment therapy; CGQ, Caregiver Guilt Questionnaire; CFQ-7, Cognitive Fusion Questionnaire-7; ELS-9, Engaged Living Scale -9; F/U, follow-up; GAD-7, Generalized Anxiety Disorder-7; MM-CGI-BF, Marwit–Meuser Caregiver Grief Inventory-Brief-Form; PHQ-9, Patient Health Questionnaire-9; PSS-10, Perceived Stress Scale -10; SCS-SF, Self-Compassion Scale-Short Form; WHOQOL‑BREF-Psych, World Health Organization Quality of Life Assessment‑BREF-Psychological Health Component; ZBI-12, Zarit Burden Interview-12.
